# Supplementary material for: Impact of systemic anticancer therapy in pediatric optic pathway glioma on visual function: A systematic review
Source: PLoS One. 2021 Oct 21;16(10):e0258548. doi: 10.1371/journal.pone.0258548 (PMC8530362; doi:10.1371/journal.pone.0258548)
Supplement: S1 File — (PDF) [file pone.0258548.s001.pdf]

To enable PROSPERO to focus on COVID-19 submissions, this registration record has undergone basic automated checks for eligibility and is published exactly as submitted. It has since been amended by the author and the PROSPERO team have checked the record for eligibility.
PROSPERO has never provided peer review, and usual checking by the PROSPERO team does not endorse content. Therefore, automatically published records should be treated as any other PROSPERO registration. Further detail is provided here.

Citation

Laura Wijninga, C. Bennebroek, P. Saeed, A.Y.N. Schouten-van Meeteren, J. Limpens. Effect of systemic anticancer therapy on visual acuity in children with optic pathway glioma (OPG): a systematic review. PROSPERO 2020 CRD42020125576 Available from:
https://www.crd.york.ac.uk/prospero/display_record.php?ID=CRD42020125576

Review question

What is the effect of treatment with systemic anticancer therapy on visual acuity in children with optic pathway glioma (OPG)?

P = children (age 0-18 yrs) with OPG, that required treatment

I = treatment with systemic anticancer therapy

C = no studies on control groups are available. Stratification in the included population will be performed on :

1. Neurofibromatosis type 1 OPG - sporadic OPG

2. anatomic location of OPG: optic nerve/ chiasm/ beyond optic nerve/ combination of 2 of 3 location

O = change in visual acuity: as no uniform consensus exists on the definition in change of visual acuity in the field of pediatric OPG, all definitions of included articles will be scored

Searches

The present study will follow the PRISMA-reporting guidelines.

An information specialist (J.L.) will search:

-Ovid MEDLINE(R) and Epub Ahead of Print, In-Process & Other Non-Indexed Citations and Daily: 1990*-2020

-Embase Classic+Embase: 1947 - 2020 August

Thesaurus terms like MeSH-terms and free text terms for [1] optic (nerve/pathway) glioma and chemotherapy/cytostatic/antineoplastic drugs or [2] a narrow search for prospective and retrospective studies on OPG management. The two searches will be combined with a pediatric search filter. Conference abstracts will be excluded. No date or language restrictions will be applied. Identified records will be imported in ENDNOTE and duplicate records will be removed.

Database(s): Ovid MEDLINE(R) and Epub Ahead of Print, In-Process & Other Non-Indexed Citations and Daily: 1990*- 2020
* Systemic anticancer for pediatric OPG is applied since 1990.

Reviews, conference abstract, editorials, notes and studies not available in full text were excluded from this review.

Types of study to be included
Prospective and retrospective studies (mono-/ multicenter) are included with a minimum volume of: N = 10

Studies which evaluated change in visual acuity (pre- versus post-systemic anticancer therapy) are included.

Condition or domain being studied
Optic Pathway Glioma (OPG) are a subgroup of pediatric low grade glioma. These tumors are specifically located in the tract of optic nerve to optic tract. Regardless of a level of overall survival, visual function is frequently seriously deprived at the start of therapy. Currently, the first choice of treatment for unresectable OPG is systemic anticancer therapy. This review will present an overview of studies that published on the effect of systemic anticancer therapy on change in visual acuity, with emphasis on the definition of change in visual acuity.

Participants/population
Studies were included: when reporting on change of visual acuity in children (? 18 year) after systemic anticancer therapy for OPG / including a minimum of 10 patients per study / reporting on patients receiving surgical treatment (biopsy/ liquor drain/ tumor resection) prior to CT.

Studies reporting on prior radiotherapy were excluded.

Intervention(s), exposure(s)
Treatment with solely first-line or following line chemotherapy. Diverse chemotherapy regimes are accepted for inclusion.

Comparator(s)/control
We expect no randomised or case controlled series exist in current literature on this topic, no control group is available.

Main outcome(s)
The primary endpoint of this review is the number and percentage of OPG patients with change of visual acuity (VA) after systemic anticancer therapy. Change is divided in 3 groups: improvement/ stabilisation/ deterioration. The secondary endpoints is change in visual field.

Concerning the definition of change in VA: As currently no consensus exist on the definition of change in visual acuity for children with OPG (or children with brain cancer), all definitions are registered and evaluated. Meta-analysis is preferably performed on a definition of change of VA ? = ? 0.2 LogMAR (Logarithm of the Minimum Angle of Resolution).

Measures of effect

Change in visual acuity divided in 3 subgroups: nrs and percentage.

Additional outcome(s)
Intention for stratification on:

1. Neurofibromatosis type 1 OPG - sporadic OPG
2. anatomic location of OPG: optic nerve/ chiasm/ beyond optic nerve/ combination of 2 of 3 location

ODDS ratio applied in the 3 subgroups of change in VA

Measures of effect

Odds ratio. A previously performed search & analysis allowed no analysis of the Odds ratio (results not published by this group), therefore no number needed to treat is calculated.

Data extraction (selection and coding)
Title, abstract and full text will be screened by two researchers. Any disagreement about inclusion will be

adjudicated by a third researcher.

Extracted data from each study: study design, name of the first author, country of study performed, year of publication, number participating patients, definition of change in visual acuity, median or mean age at or start of treatment, localization of OPG according to (Modified) Dodge Classification, ratio Neurofibromatosis type 1/ sporadic OPG, follow up period, chemotherapy regimen, decrease/ stability/ increase of VA in time interval start to end of follow up, prognostic factors for decrease of VA.

Risk of bias (quality) assessment

Two researchers will screen the abstracts independently. Any disagreement about whether a study should be included will be adjudicated by a third researcher.

As the majority of studies on OPG and visual outcome are not RCTs, we expect that most studies will be qualitative. Therefore the Checklist for Case Series- The Joanna Briggs Institute Critical Appraisal tools for use in JBI Systematic Reviews- will be used to evaluate the level of evidence of included studies.

Strategy for data synthesis

Studies are synthesized through a structured narrative review of results of included studies. A summary-of-findings table will be included. When appropriate and where suitable data are available, meta-analysis will be employed to estimate a summary measure of effect on relevant outcomes. The specific methods for meta-analysis and for the detection and investigation of heterogeneity will depend upon the summary measure selected and will used standard procedures recommended by the Cochrane Collaboration. Cochrane Review Manager (RevMan 5.3) software will be used to perform meta-analysis. Heterogeneity will be explored through consideration of the study populations methods and interventions, by visualisation of results and, in statistical terms, by the $?^2$ test for homogeneity and the I^2 statistic.

Analysis of subgroups or subsets

Synthesis regarding the main outcome will be described in change in visual acuity.

Intention for stratification on:

1. Neurofibromatosis type 1 OPG - sporadic OPG

2. anatomic location of OPG: optic nerve/ chiasm/ beyond optic nerve/ combination of 2 of 3 location

Odds ratio applied in the 3 subgroups of change in VA

Contact details for further information

Laura Wijninga
l.e.wijninga@amc.uva.nl

Organisational affiliation of the review

Department of Ophthalmology, Amsterdam UMC

Review team members and their organisational affiliations

Miss Laura Wijninga. Amsterdam UMC
Dr C. Bennebroek. Amsterdam UMC
Dr P. Saeed. Amsterdam UMC
Dr A.Y.N. Schouten-van Meeteren. Princes Màxima Centrum, The Netherlands
Dr J. Limpens. Amsterdam UMC

Type and method of review

Intervention, Systematic review

Anticipated or actual start date

13 March 2020

Anticipated completion date

30 October 2020

Funding sources/sponsors

This review will be initiated and performed by a medical student (LW) and PHD student/ pediatric ophthalmologist (CB), both affiliated to the Amsterdam University Medical Center. This process will be performed without funding or sponsoring

Conflicts of interest

Language

English

Country

Netherlands

Stage of review

Review Ongoing

Subject index terms status

Subject indexing assigned by CRD

Subject index terms

Child; Humans; Neurofibromatosis 1; Optic Nerve Glioma

Date of registration in PROSPERO

28 April 2020

Date of first submission

12 March 2019

Details of any existing review of the same topic by the same authors

This version is considered an update of the protocol due to the following reasons:

-the intended search period: 2008-2020 revealed a paucity of studies rendering stratification impossible, therefore the period of search was extended to the start of application of systemic anticancer therapy to 2020.

-Due to to a change in terminology of OPG, an information specialist has performed the current search (J.L.)

Stage of review at time of this submission

Stage	Started	Completed
Preliminary searches	Yes	No
Piloting of the study selection process	Yes	No
Formal screening of search results against eligibility criteria	Yes	No
Data extraction	No	No
Risk of bias (quality) assessment	No	No
Data analysis	No	No

Revision note

This version is considered an update of the protocol due to the following reasons:-the intended search period: 2008-2020 revealed a paucity of studies rendering stratification impossible, therefore the period of search was extended to the start of application of systemic anticancer therapy to 2020.-Due to to a change in

terminology of OPG, an information specialist has performed the current search (J.L.)

The record owner confirms that the information they have supplied for this submission is accurate and complete and they understand that deliberate provision of inaccurate information or omission of data may be construed as scientific misconduct.
The record owner confirms that they will update the status of the review when it is completed and will add publication details in due course.

Versions

28 April 2020
26 January 2021
