# Supplementary material for: Impact of systemic anticancer therapy in pediatric optic pathway glioma on visual function: A systematic review
Source: PLoS One. 2021 Oct 21;16(10):e0258548. doi: 10.1371/journal.pone.0258548 (PMC8530362; doi:10.1371/journal.pone.0258548)
Supplement: S1 Table — Search strategy Ovid MEDLINE(R) and Epub Ahead of Print, In-Process & Other Non-Indexed Citations and Daily 1946 to August 04, 2020. Date of search: 2020-08-06. (DOCX) [file pone.0258548.s003.docx]

**S1 Table. Search strategy Ovid MEDLINE(R)and Epub Ahead of Print.**

Search strategy Ovid MEDLINE(R) and Epub Ahead of Print, In-Process & Other Non-Indexed Citations and Daily 1946 to August 04, 2020. Date search: 2020-08-06

| **#** | **Searches** | **Results** |
| --- | --- | --- |
| **1** | optic nerve glioma/ | **457** |
| **2** | (glioma/ or astrocytoma/) and (optic tract/ or optic nerve/ or optic chiasm/ or optic nerve neoplasms/) | **817** |
| **3** | *optic nerve neoplasms/dt | **29** |
| **4** | ((((optic or visual) adj2 (pathway* or tract*)) or (optic adj2 (thalam* or hypothala* or prechias*)) or postchias* or chias*) and (gliom* or astrocytom*)).tw,kf. | **1295** |
| **5** | (ONG or ONGs or OPG or OPGs or OPHG or OPHGs or OCHG* or OHPA or OHPAs).tw,kf. and (gliom* or astrocytom* or ((nerve or optic) adj3 tum*)).mp. | **205** |
| **6** | (optic nerve adj (gliom* or astrocytom*)).tw,kf. | **368** |
| **7** | optic glioma*.tw,kf. | **479** |
| **8** | (((low-grade adj3 (glioma or gliomas or astrocytom*)) or pilocytic astrocytom*).tw,kf. or ((LGG or LGGs or PLGG or PLGGs).tw,kf. and gliom*.mp.)) and (vision or hypovision* or visual or visually or optic or VA or blind* or ((partial* or impair*) adj2 sight*) or op?thalmologic*).mp. | **709** |
| **9** | (((((optic or visual) adj2 (pathway or tract* or chias* or prechias* or postchias* or hypothal* or thalam*)) or (chiasm* adj1 (hypothal* or thalam*))) adj3 (tumo?r* or neoplasm*)) or optic nerve tumo?rs*).ti. | **102** |
| **10** | or/1-9 [OPGs / OPT as major topic] | **2648** |
| **11** | antineoplastic combined chemotherapy protocols/ | **140355** |
| **12** | carboplatin/ or cisplatin/ or procarbazine/ or antineoplastic agents, phytogenic/ or etoposide/ or vinblastine/ or vincristine/ or antibiotics, antineoplastic/ or doxorubicin/ or antimetabolites, antineoplastic/ or fluorouracil/ or thioguanine/ or antineoplastic agents, alkylating/ or cyclophosphamide/ or dacarbazine/ or mitolactol/ or nitrosourea compounds/ or carmustine/ or lomustine/ or hydroxyurea/ | **268552** |
| **13** | (cystostat* or chemother* or chemo-ther* or polychemother* or multichemother*).tw,kf. | **415714** |
| **14** | (carboplatin* or cisplatin* or cis-platin* or cis-diamminedichloroplat* or CDDP or CisPt or Cis-Pt or procarbazin* or etoposi* or eposid* or VP-16 or VP16 or vinca alkaloid* or vinblastin* or vincristin* or vinorelbin* or doxorubicin* or antimetabolit* or anti-metabolit* or fluorouracil* or 5FU or 5-FU or t?ioguanin* or alkylating drug* or cyclophos* or CY or d#carbazin* or DTIC or ICDT or mitolactol* or dibromodulcitol* or NSC-104800 or NSC104800 or nitroso* or carmustin* or BCNU or BiCNU or lomustin* or CCNU or CeeNU or hydroxyurea* or hydrea hydroxycarbamid* or oncocarbamid*).tw,kf. | **268044** |
| **15** | (SFOP or BBSFOP or TPDCV).tw,kf. | **91** |
| **16** | or/11-15 [ chemotherapy ] | **703973** |
| **17** | 10 and 16 [ I OPG + chemotherapy ] | **382** |
| **18** | *antineoplastic agents/ or molecular targeted therapy/ | **221049** |
| **19** | angiogenesis inhibitors/ or antibodies, monoclonal, humanized/ or "antineoplastic agents, immunological"/ or bevacizumab/ | **65656** |
| **20** | protein kinase inhibitors/ or exp *protein kinases/ai or exp proto-oncogene proteins/ai or benzimidazoles/ | **100026** |
| **21** | (trametinib or AZD 6244 or dabrafenib).rn. | **1229** |
| **22** | ((antineoplast* or anti-neoplast* or anticancer or anti-cancer or antitumo?r or anti-tumo?r) adj3 (agent* or drug* or therap* or treatment* or biological*)).tw,kf. | **117216** |
| **23** | (molec* adj1 target* adj2 (therap* or treat* or drug* or agent* or medicin*)).tw,kf. | **10840** |
| **24** | (((angiogene* or vascular endothelial growth factor* or VEGF*) adj3 (inhibit* or anti or block* or antagonist* or targeting or targeted)) or antiangiogene* or antiVEGF or (humanized adj2 (Ab or Abs or Moab* or monoclonal* or antibod*)) or bevacizumab or BVZ or Avastin or Mvasi).tw,kf. | **54297** |
| **25** | (((protein kinas* or BRAF* or MEK* or MAPK* or MAP-kinase) adj3 (inhibit* or anti or antagonist* or block* or targeting or targeted)) or trametinib or mekinist or JTP74057 or JTP-74057 or GSK1120212 or GSK-1120212 or selumetinib or ZD6244 or ZD-6244 or AZD6244 or AZD-6244 or ARRY142886 or ARRY-142886 or dabrafenib or GSK2118436 or GSK-2118436).tw,kf. | **52358** |
| **26** | or/18-25 [ antineoplastic agents, VEGF- & protein kinase inhibitors ] | **499911** |
| **27** | 10 and 26 [ II OPG and neoplastic agents, VEGF & protein kinase inhibitors ] | **78** |
| **28** | ((optic pathway gliom* or optic nerve gliom*).ti. or *optic nerve glioma/) and (child* or infant* or p?ediatric).ti. and (vision or hypovision* or visual or visually or VA or blind* or ((partial* or impair*) adj2 sight*) or op?thalmologic*).mp. and (management.ti. or (treatment or therapy).tw. or dt.fs.) and ((exp case-control studies/ or exp cohort studies/ or cross-sectional studies/ or registries/ or (cohort or prospectiv* or cross*ection* or cross-section* or retrospect* or registry* or registries).tw,kf.) not (editorial/ or review/ or (review or cochrane).ti.)) [ III cohort studies on management pediatric OPG (major topic) ] | **33** |
| **29** | 17 or 27 or 28 [ I II III chemotherapy/other antineoplastic agents/ management OPG ] | **427** |
| **30** | (exp child/ or exp infant/ or (p?ediatric* or child or children* or childhood or infant* or infanc* or neonat* or neo-nat* or newborn* or new-born* or baby or babies or toddler* or prekindergarten* or kindergarten* or preschool* or school-age* or schoolage* or puber* or teens or teenager* or youth or juvenile* or boys or girls).tw,kf.) not (exp animals/ not humans/) [ pediatric filter ] | **3143257** |
| **31** | 29 and 30 [ I II human pediatric OPG + chemotherapy / management ] | **347** |
| **32** | limit 31 to yr="1990 -Current" [ I II III pediatric OPG therapy >1990 ] | **336** |
| **33** | remove duplicates from 32 [ I II III human pediatric OPG + chemotherapy / management >1990 - deduplicated ] | **336** |
