# Supplementary material for: Impact of systemic anticancer therapy in pediatric optic pathway glioma on visual function: A systematic review
Source: PLoS One. 2021 Oct 21;16(10):e0258548. doi: 10.1371/journal.pone.0258548 (PMC8530362; doi:10.1371/journal.pone.0258548)
Supplement: S2 Table — Search strategy Embase Classic+Embase 1947 to 2020 August 05. Date of search: 2020-08-06. (DOCX) [file pone.0258548.s004.docx]

**S2 Table. Search Strategy Embase Classic + Embase**

Search Strategy Embase Classic+Embase 1947 to 2020 August 05. Date of search: 2020-08-06

| **#** | **Searches** | **Results** |
| --- | --- | --- |
| **1** | optic nerve glioma/ or pilocytic astrocytoma/ or (optic pathway adj2 glioma*).dq. | **4515** |
| **2** | (glioma/ or astrocytoma/) and (optic tract/ or optic nerve/ or optic chiasm/ or optic nerve neoplasms/) | **1229** |
| **3** | *optic nerve neoplasms/dt or (*glioma/dt and cancer grading/ and preschool child/) | **72** |
| **4** | ((((optic or visual) adj2 (pathway* or tract*)) or (optic adj2 (thalam* or hypothala* or prechias*)) or postchias* or chias*) and (gliom* or astrocytom*)).tw,kw. | **2173** |
| **5** | (ONG or ONGs or OPG or OPGs or OPHG or OPHGs or OCHG* or OHPA or OHPAs).tw,kw. and (gliom* or astrocytom* or ((nerve or optic) adj3 tum*)).mp. | **402** |
| **6** | (optic nerve adj (gliom* or astrocytom*)).tw,kw. | **590** |
| **7** | optic glioma*.tw,kw. | **774** |
| **8** | (((low-grade adj3 (glioma or gliomas or astrocytom*)) or pilocytic astrocytom*).tw,kw. or ((LGG or LGGs or PLGG or PLGGs).tw,kw. and gliom*.mp.)) and (vision or hypovision* or visual or visually or optic or VA or blind* or ((partial* or impair*) adj2 sight*) or op?thalmologic*).mp. | **1448** |
| **9** | (((((optic or visual) adj2 (pathway or tract* or chias* or prechias* or postchias* or hypothal* or thalam*)) or (chiasm* adj1 (hypothal* or thalam*))) adj3 (tumo?r* or neoplasm*)) or optic nerve tumo?rs*).ti. | **131** |
| **10** | or/1-9 [OPGs / OPT as major topic] | **7234** |
| **11** | cancer chemotherapy/ or chemotherapy/ or combination chemotherapy/ or cancer combination chemotherapy/ or multimodal chemotherapy/ or chemosensitivity/ | **542725** |
| **12** | *alkylating agent/ or carboplatin/ or cisplatin/ or dacarbazine/ or nitrosourea derivative/ or carmustine/ or lomustine/ or procarbazine/ or cyclophosphamide/ or mitolactol/ or *antineoplastic alkaloid/ or etoposide/ or vinblastine/ or vincristine/ or *anthracycline antibiotic agent/ or doxorubicin/ or *antineoplastic antimetabolite/ or fluorouracil/ or tioguanine/ or hydroxyurea/ | **648522** |
| **13** | (cystostat* or chemother* or chemo-ther* or polychemother* or multichemother*).tw,kw. | **673278** |
| **14** | (carboplatin* or cisplatin* or cis-platin* or cis-diamminedichloroplat* or CDDP or CisPt or Cis-Pt or procarbazin* or etoposi* or eposid* or VP-16 or VP16 or vinca alkaloid* or vinblastin* or vincristin* or vinorelbin* or doxorubicin* or antimetabolit* or anti-metabolit* or fluorouracil* or 5FU or 5-FU or t?ioguanin* or alkylating drug* or cyclophos* or CY or d#carbazin* or DTIC or ICDT or mitolactol* or dibromodulcitol* or NSC-104800 or NSC104800 or nitroso* or carmustin* or BCNU or BiCNU or lomustin* or CCNU or CeeNU or hydroxyurea* or hydrea hydroxycarbamid* or oncocarbamid*).tw,kw. | **388155** |
| **15** | (SFOP or BBSFOP or TPDCV).tw,kw. | **123** |
| **16** | or/11-15 [ chemotherapy ] | **1269706** |
| **17** | 10 and 16 [ I OPG + chemotherapy ] | **1549** |
| **18** | *antineoplastic agents/ | **117642** |
| **19** | *molecularly targeted therapy/ or (molecularly targeted therapy/ and (mapk signaling/ or vegf signaling/ or exp protein serine threonine kinase/)) | **15785** |
| **20** | exp *angiogenesis inhibitor/ or immunological antineoplastic agent/ or antineoplastic monoclonal antibody/ or bevacizumab/ | **145946** |
| **21** | exp *protein serine threonine kinase inhibitor/ae, ad, cb, cm, ct, dt, iv, po or exp *protein tyrosine kinase inhibitor/ae, ad, cb, cm, ct, dt, iv, po or b raf kinase inhibitor/ or *dabrafenib/ or exp mitogen activated protein kinase kinase inhibitor/ [ incl. trametinib & selumetinib ] | **109293** |
| **22** | ((antineoplast* or anti-neoplast* or anticancer or anti-cancer or antitumo?r or anti-tumo?r) adj3 (agent* or drug* or therap* or treatment* or biological*)).tw,kw. | **154326** |
| **23** | (molec* adj1 target* adj2 (therap* or treat* or drug* or agent* or medicin*)).tw,kw. | **16647** |
| **24** | (((angiogene* or vascular endothelial growth factor* or VEGF*) adj3 (inhibit* or anti or block* or antagonist* or targeting or targeted)) or antiangiogene* or antiVEGF or (humanized adj2 (Ab or Abs or Moab* or monoclonal* or antibod*)) or bevacizumab or BVZ or Avastin or Mvasi).tw,kw. | **88233** |
| **25** | (((protein kinas* or BRAF* or MEK* or MAPK* or MAP-kinase) adj3 (inhibit* or anti or antagonist* or block* or targeting or targeted)) or trametinib or mekinist or JTP74057 or JTP-74057 or GSK1120212 or GSK-1120212 or selumetinib or ZD6244 or ZD-6244 or AZD6244 or AZD-6244 or ARRY142886 or ARRY-142886 or dabrafenib or GSK2118436 or GSK-2118436).tw,kw. | **67930** |
| **26** | or/18-25 [ antineoplastic agents, VEGF- & protein kinase inhibitors ] | **571352** |
| **27** | 10 and 26 [ II OPG and antineoplastic agents, VEGF- & protein kinase inhibitors ] | **453** |
| **28** | ((optic pathway gliom* or optic nerve gliom*).ti. or *optic nerve glioma/ or *pilocytic astrocytoma/) and (child* or infant* or p?ediatric).ti. and (vision or hypovision* or visual or visually or VA or blind* or ((partial* or impair*) adj2 sight*) or op?thalmologic*).mp. and (management.ti. or (treatment or therapy).tw. or dt.fs.) and ((exp case control study/ or cohort analysis/ or follow up/ or longitudinal study/ or prospective study/ or retrospective study/ or cross-sectional study/ or register/ or (cohort or prospectiv* or cross*ection* or cross-section* or retrospect* or registry* or registries).tw,kw.) not (review.pt. or review.ti.)) [ III cohort studies on management pediatric OPG (major topic)] | **114** |
| **29** | 17 or 27 or 28 [ I II III chemotherapy/other antineoplastic agents/ management OPG ] | **1768** |
| **30** | (child/ or preschool child/ or school child/ or toddler/ or childhood cancer/ or (p?ediatric* or child or children* or childhood or infant* or infanc* or neonat* or neo-nat* or newborn* or new-born* or baby or babies or toddler* or prekindergarten* or kindergarten* or preschool* or school-age* or schoolage* or puber* or teens or teenager* or youth or juvenile* or boys or girls).tw,kw.) not (exp animal/ not human/) | **3623988** |
| **31** | 29 and 30 [ I II III human pediatric OPG + chemotherapy / management ] | **1356** |
| **32** | editorial/ or (editorial or note or conference abstract or conference review).pt. | **5366106** |
| **33** | 31 not 32 [ I II human pediatric OPG + chemotherapy / management - not conference abstracts, editorials, notes ] | **828** |
| **34** | limit 33 to yr="1990 -Current" [ I II III pediatric OPG therapy >1990 ] | **806** |
| **35** | 34 not medline.cr. [ I II III pediatric OPG therapy >1990, embase records only] | **769** |
| **36** | remove duplicates from 35 [ I II III pediatric OPG therapy >1990, embase records only - deduplicated] | **765** |
