# Supplementary material for: Impact of systemic anticancer therapy in pediatric optic pathway glioma on visual function: A systematic review
Source: PLoS One. 2021 Oct 21;16(10):e0258548. doi: 10.1371/journal.pone.0258548 (PMC8530362; doi:10.1371/journal.pone.0258548)
Supplement: S3 Table — (DOCX) [file pone.0258548.s005.docx]

**S3 Table. JBI-Critical appraisal of included case series.**

Light grey boxes: low risk of bias

| **Author/**  **JBI-CA assessment** | **1: Clear criteria for inclusion?** | **2: Condition measured in standard, reliable way?** | **3: Valid methods for identification of condition?** | **4: Consecutive inclusion participants?** | **5: Complete inclusion participants?** | **6: Clear reporting demographics participants?** | **7: Clear reporting clinical information participants?** | **8: Outcome follow up results clearly reported?** | **9: Clear reporting presenting site/demographics?** | **10: Statistical analysis appropriate?** |  | **Cumulative response**  **per study** | | | | |
| --- | --- | --- | --- | --- | --- | --- | --- | --- | --- | --- | --- | --- | --- | --- | --- | --- |
|  |  |  |  |  |  |  |  |  |  |  |  | **Yes** | | **No** | | **Unsure** |
| **Massimino et al. (2002) ^9^** | Y | U | Y | U | U | Y | N | N | N | U |  | 3 | 3 | | 4 | |
| **Dalla Via et al. (2007) ^36^** | Y | Y | Y | Y | Y | Y | Y | N | Y | U |  | 8 | 1 | | 1 | |
| **Massimino et al. (2010) ^37^** | Y | U | Y | U | U | Y | N | N | N | U |  | 3 | 4 | | 3 | |
| **Shofty et al. (2011) ^39^** | Y | Y | Y | Y | Y | Y | N | N | N | Y |  | 7 | 3 | | 0 | |
| **Fisher et al. (2012) ^23^** | Y | Y | Y | U | U | Y | Y | Y | U | U |  | 6 | 0 | | 4 | |
| **Kalin-Hadju et al.(2014) ^24^** | Y | N | N | U | U | Y | Y | Y | Y | U |  | 5 | 2 | | 3 | |
| **Dodgshun et al. (2015) ^35^** | Y | N | Y | U | Y | Y | Y | U | Y | Y |  | 7 | 1 | | 2 | |
| **Prada et al (2015) ^45^** | Y | U | U | Y | Y | Y | N | N | N | U |  | 4 | 3 | | 3 | |
| **Doganis et al.(2016) ^38^** | U | Y | U | Y | Y | U | N | Y | N | U |  | 4 | 2 | | 4 | |
| **Lassaletta et al. (2016) ^11^** | Y | U | Y | U | Y | Y | N | N | N | U |  | 4 | 3 | | 3 | |
| **Falzon et al. (2018) ^22^** | Y | Y | Y | Y | U | Y | Y | Y | Y | Y |  | 9 | 0 | | 1 | |
