# Supplementary material for: Impact of systemic anticancer therapy in pediatric optic pathway glioma on visual function: A systematic review
Source: PLoS One. 2021 Oct 21;16(10):e0258548. doi: 10.1371/journal.pone.0258548 (PMC8530362; doi:10.1371/journal.pone.0258548)
Supplement: Attachment — Submitted filename: Response to reviewers-PLOS ONE-30082021.docx.pdf [file pone.0258548.s006.pdf]

Editor-in-Chief: **Dr J. Heber**
PLOS ONE

August 30, 2021

Dear Dr Heber,

**Subject: Response to reviewers**

I herewith send our response to the reviewers received on August 2, 2021, regarding our manuscript entitled *'Impact of systemic anticancer therapy in pediatric optic pathway glioma on visual function: a systematic review'* (PONE-D-21-14820).

Concerning point 1, 2 and 3 we have received no comments from the reviewers. We kindly thank you for your review.

4. Is the manuscript presented in an intelligible fashion and written in standard English?

    Reviewer #1: Yes

    Reviewer #2: No

    *Response to reviewer 2: The manuscript has been revised for syntax and grammar by a primary English speaker who is a professional editor. (Editing declaration attached.)*

5. Review Comments to the Author

Reviewer #1: This is a well crafted review of SAT in OPG on visual function. This review highlights the need for prospective studies, as the authors point out, with more uniform parameters. The hope with this type of publication is that future studies will use for uniform methods of VF analysis.

*Response to reviewer 1: We thank you for your comments.*

Reviewer #2: I don't think the results are earth-shattering, but it is important to point out that the outcomes regarding visual improvement in OPG chemotherapeutic treatment is inconsistent.

*Response to reviewer 2: The manuscript has been fully revised for syntax and grammar by a primary English speaker (see: OPG and SAT-PLOS ONE-revisited-track changes-25082021)*

The manuscript has been adjusted to meet *PLOS ONE*'s style requirements.

We highly appreciate the suggestions made by the reviewers and we are looking forward to your decision on the suitability of our manuscript for publication in *PLOS ONE.*

Sincerely yours,

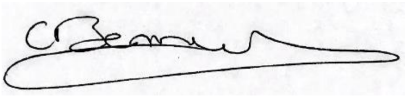

Carlien Bennebroek, MD

Pediatric Ophthalmologist
Department of Ophthalmology, Amsterdam UMC
Meibergdreef 9 D2-422
Postbus 22660
1100 DD, Amsterdam, The Netherlands
c.a.bennebroek@amsterdamumc.nl
Tel: +31 626096193
Fax: +31 205669078
